# A Treatise on Diseases of the Heart

**Published:** 1853-11

**Authors:** 


ART. III.—*A Treatise on Diseases of the Heart.* By O'B. BELLINGHAM, M. D., F. R. C. S., Member of the Court of Examiners of the Royal College of Surgeons in Ireland, one of the Medical Officers of St. Vincent's Hospital, and one of the Secretaries of the Surgical Society of Ireland. Dublin: Fannin & Co.

It is, we presume, to the kindness of the editor of the Dublin Medical Press, that we are indebted for the first part of this very excellent treatise. It is coming to be a well understood matter, that monographs on single organs and their diseases, constitute the most valuable additions to medical literature. Works on practice, though useful, can never cover the whole ground of special subjects. Of this latter class only Watson's Practice has attained to any lasting reputation in recent times.

The advantages of monographs are easily perceived. The ample pages of Bennet on Inflammation of the Uterus, exhaust the subject, and bring the reader to a full appreciation of that specialty in all its bearings. Dr. Bellingham, in this work, attempts to accomplish the same for the Heart, and its diseases. In the first part he considers the anatomy of the heart, as necessary to a proper appreciation of its functions. This portion of the work is full, and the style of description is easy and readable. It contains a description of its relations to surrounding organs, its size, weight, and measurements, with all necessary details concerning its motions and sounds.

The remainder of the first part is devoted to the examination of the heart in disease, its physical signs, and the general signs with the remote effects of cardiac disease.

The second part, (which we hope soon to receive,) is to be given to the consideration of the individual diseases of the heart. The work is statistical in character, and Dr. Bellingham has necessarily availed himself to a large extent of the researches of others. The dedication is a graceful tribute to the worth of Dr. Arthur Jacob, the editor of the Dublin Medical Press. Altogether it is an interesting and valuable production, and we hope soon to announce its republication on this side the Atlantic, trusting, also, that the present neat and elegant typography may be preserved. S. B. H.

